# Thermally Conductive and UV-EMI Shielding Electronic Textiles for Unrestricted and Multifaceted Health Monitoring

**DOI:** 10.1007/s40820-024-01429-x

**Published:** 2024-05-21

**Authors:** Yidong Peng, Jiancheng Dong, Jiayan Long, Yuxi Zhang, Xinwei Tang, Xi Lin, Haoran Liu, Tuoqi Liu, Wei Fan, Tianxi Liu, Yunpeng Huang

**Affiliations:** Key Laboratory of Synthetic and Biological Colloids, Ministry of Education, School of Chemical and Material Engineering, Jiangnan University, Wuxi, 214122 People’s Republic of China

**Keywords:** Skin electronics, Thermal regulating textiles, Electromagnetic interference shielding, Ultraviolet proof, Health monitoring

## Abstract

Skin-attachable electronics have garnered considerable research attention in health monitoring and artificial intelligence domains, whereas susceptibility to electromagnetic interference (EMI), heat accumulation issues, and ultraviolet (UV)-induced aging problems pose significant constraints on their potential applications. Here, an ultra-elastic, highly breathable, and thermal-comfortable epidermal sensor with exceptional UV-EMI shielding performance and remarkable thermal conductivity is developed for high-fidelity monitoring of multiple human electrophysiological signals. Via filling the elastomeric microfibers with thermally conductive boron nitride nanoparticles and bridging the insulating fiber interfaces by plating Ag nanoparticles (NPs), an interwoven thermal conducting fiber network (0.72 W m^−1^ K^−1^) is constructed benefiting from the seamless thermal interfaces, facilitating unimpeded heat dissipation for comfort skin wearing. More excitingly, the elastomeric fiber substrates simultaneously achieve outstanding UV protection (UPF = 143.1) and EMI shielding (SE_*T*_ > 65, X-band) capabilities owing to the high electrical conductivity and surface plasmon resonance of Ag NPs. Furthermore, an electronic textile prepared by printing liquid metal on the UV-EMI shielding and thermally conductive nonwoven textile is finally utilized as an advanced epidermal sensor, which succeeds in monitoring different electrophysiological signals under vigorous electromagnetic interference. This research paves the way for developing protective and environmentally adaptive epidermal electronics for next-generation health regulation.

**Supplementary Information:**

The online version contains supplementary material available at 10.1007/s40820-024-01429-x.

## Highlights

- Ag nanoparticles are evenly plated on BN-embedded fibers as a thermally conductive sheath, bridging the insulating interface between fibers to construct a 3D heat transfer network.
- The LM-printed e-textile shows outstanding capability for the monitoring of human ECG, sEMG, and EEG signals even under intense EM interference, when commercial electrodes cannot work properly.
- The e-textile simultaneously manifests excellent EMI shielding (SET > 65, X-band) and UV protection (UPF = 143.1) performance, thus protecting the device and skin from harmful radiation.

**Supplementary Information:**

The online version contains supplementary material available at 10.1007/s40820-024-01429-x.

## Graphical abstract

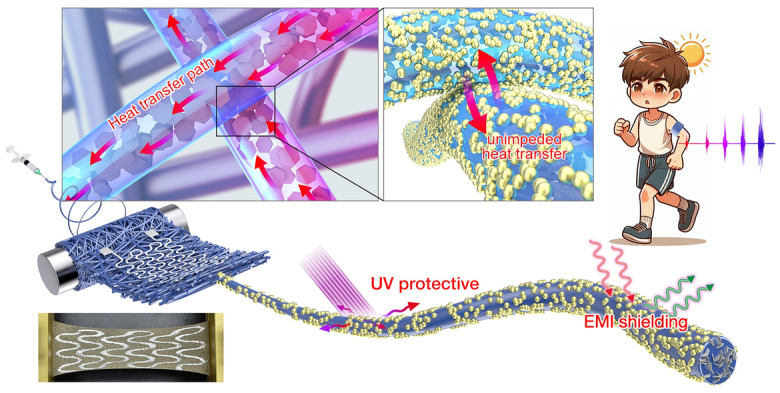

## Introduction

Flexible electronics have recently witnessed vast progress in the fields of electronic skins [1–5], soft robotics [6–9], human–machine interfaces [10–13] and medical devices [14–17]. Among them, electronic textiles (e-textiles), with features including moisture-permeability, skin-friendliness, and stable electrical/mechanical characteristics, have shown substantial potential in health monitoring as epidermal sensors [18–22]. Generally, e-textiles must consist of integrated flexible circuits and stretchable textile substrates. In terms of flexible circuit materials, gallium-indium eutectic-based liquid metals (LM) are considered outstanding candidates for their unlimited ductility, high conductivity, and favorable biocompatibility [23–25]. Stretchable textile substrates should be air/moisture permeable to ensure long-term wearing comfort. In addition, the thermal dissipation performance of e-textiles cannot be ignored, most polymer substrates (i.e., thermoplastic polyurethanes (TPU), polydimethylsiloxane (PDMS)) are characterized by low thermal conductivity, and thus heat will be trapped inside the devices and failed being dissipated to the surroundings [26]. Besides, heat in electronic textiles can be obtained from the external environment (i.e., light and hot air). The above two factors may significantly increase the temperature on skin surfaces and devices, causing the deterioration of electrical and mechanical properties, shortening the service lifetime, and even causing serious thermal damage to human skin. Therefore, heat dissipation problems should be considered during the development of e-textile devices, which have not yet received enough research attention.

To mitigate e-textile devices from overheating, traditional solutions are to apply coolant or thermoelectric materials through heat conduction and convection. However, these additional cooling systems require excessive space and power supply, compromising the portability and comfort of wearable devices. Incorporating thermally conductive fillers into polymer substrates to improve thermal conductivity without affecting wearability is an option worth considering [27, 28]. There are three main types of thermally conductive fillers, metal-based materials (e.g., Au, Ag, and Al) [29], carbon-based materials (e.g., graphene, carbon and nanotube) [30–32], and ceramic materials (e.g., silicon carbide and aluminum nitride) [33–35]. However, metal-based fillers have drawbacks such as high density and high thermal expansion coefficient [36, 37]. Some researchers have added radially orientated carbon fibers (CFs) to improve thermal conductivity [38]. Whereas, there is a problem of high variability in thermal conductivity along different directions and poor mechanical properties. Boron nitride (BN) particles as one typical ceramic material show excellent promise for thermally conductive composites, featuring low coefficient of expansion and cost efficiency [28, 35, 39]. However, interface issues between the inorganic material and the polymer substrates, and mechanical degradation problems during hybridization still challenge the overall performance of thermally conductive fibers and textiles.

Meanwhile, e-textiles adhered to human skin must provide sufficient protection against environmental harmful effects (such as electromagnetic waves (EMW) from electrical equipment, UV radiation from the sun). Specifically, overexposure to UV radiation during the daytime can lead to sunburn, skin allergy, and even skin cancer [40]. In addition, electronic devices are inevitably affected by EMW when operated under complex electromagnetic environments [41–43]. Electromagnetic waves generated by chips and circuits can seriously affect the sensing and transmitting stability of wearable electronics and also threaten human health [44–46]. Therefore, e-textiles integrating excellent electromagnetic protection and UV-proof functionalities are of great value in daily wearable and multi-environmental adaptive electronics, which have been under-exploited in recent years.

Herein, an ultra-elastic, highly breathable, and thermal-comfortable epidermal sensor with superior UV-EMI shielding performance and excellent thermal conductivity is developed for high-fidelity monitoring of multiple human electrophysiological signals. To achieve this, an integrated thermal conducting fiber network is constructed by filling the elastomeric microfibers with BN nanoparticles (NPs) and bridging the insulating fiber interfaces via Ag NPs plating, thus significantly improving the thermal conductivity of the fiber substrates to 0.72 W m^−1^ K^−1^. More interestingly, the plating of Ag NPs provides the elastomeric fiber substrates with excellent EMI shielding (SE_*T*_ > 65, X-band) and UV protection (UPF = 143.1) performance. Apart from the efficient thermal conductivity and UV-EMI shielding, the e-textile prepared by printing liquid metal enables the monitoring of various electrophysiological signals (electrocardiograph (ECG), surface electromyogram (sEMG), and electroencephalograph (EEG)) even under vigorous electromagnetic interference. This work opens up an exciting research avenue for developing advanced epidermal sensors.

## Experimental Section

### Materials

Styrene-ethylene-butylene-styrene (SEBS) was obtained from Kraton Co., Ltd. Gallium-indium eutectic (EGaIn, Ga/In = 75.5%/24.5%) was supplied by Dongguan Huatai Metal Material Technology Co., Ltd. Boron nitride nanoparticles (BN NPs, 99.0%, particle size ≤ 4 μm) were purchased from Liaoning TanYun Aviation Material Co., Ltd. FC-4430 fluorosurfactant was supplied by Mudu Technology. Silver trifluoroacetate and laboratory solvents were all supplied by Sinopharm Co., Ltd.

### Fabrication of the UV-EMI Shielding and Thermally Conductive E-Textile

Firstly, a mixture solvent of trichloromethane and toluene (90/10 wt%) was prepared, and then BN NPs (with different weight percentages of 20, 40, 60, and 80 wt%) and FC-4430 (20 wt% to BN) were added to the above solvent under stirring to obtain FC-4430 functionalized BN NPs (FCBN). Afterward, the SEBS master batch was dissolved in the above solution (15 wt% to the total weight) under vigorous stirring to prepare the FCBN/SEBS electrospinning solution. FCBN/SEBS nonwoven textile was obtained by a versatile electrospinning process as previously reported [47–49].

For the plating of Ag nanoparticles, an ethanol solvent of silver trifluoroacetate (15 wt%) and an aqueous solvent of hydrazine hydrate (50 wt%) were first prepared. The above FCBN/SEBS textile was then soaked in the silver trifluoroacetic solution under 30 min of sonication, which was subsequently dried in the oven and placed in the hydrazine hydrate solution. After chemical reduction for 30 min, UV-EMI shielding and thermally conductive textile (denoted as AFBS) were produced. Finally, an AFBS-based e-textile was obtained by printing EGaIn and pre-stretch activation.

### Characterizations

Morphologies of the samples were observed with a S-4800 field emission scanning electron microscope (FE-SEM). The distribution of elements on the samples was determined by EDS Mapping. X-ray diffraction (XRD) analyses were carried out using a Bruker AXS D2 PHASER, Germany. Keyence VK-X150 laser microscope was used to record the 3D surface topography of samples. A universal UTM2203 tensile testing machine (Sun Technology Co., Ltd.) was used for mechanical tests. Water permeability tests were carried out using the W3-060 Water Vapor Transmission Rate Test System (30, 35, and 40 °C, 80 RH%). Water contact angles were recorded by a video optical contact angle meter (OCA15EC). UV analyses were carried out using an ultraviolet–visible spectrophotometer (Shimadzu UV-2700), Japan. ATR-FTIR spectroscopy was performed with a Thermofisher Nicolet iS50 FTIR. Electromagnetic shielding properties were studied utilizing a Keysight N5234B PNA-L Network Analyzer using a 2-port network analyzer in the range of 8.2–12.4 GHz (X-band). Electrophysiological signals of human were collected with a commercial biosignal collection kit (Plux Wireless Biosignals).

## Results and Discussion

### Design and Characterization of the Nonwoven AFBS E-Textile

**Fig. 1:**
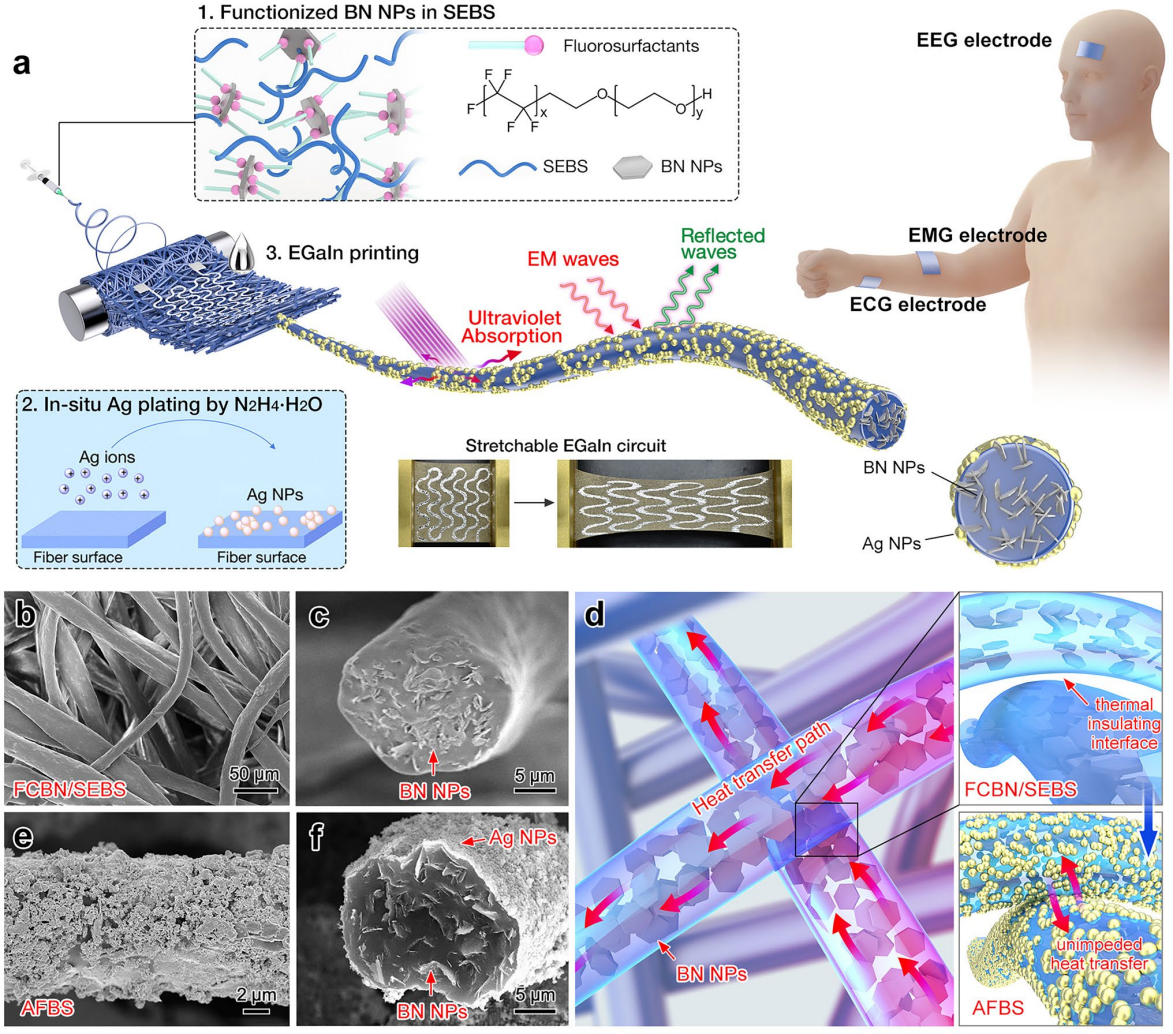
**a** Schematic illustration on the fabrication of the UV-EMI shielding and thermally conductive e-textile. **b, c** SEM images of the FCBN/SEBS microfibers embedded with BN NPs. **d** Illustration of the thermal conductive pathway in FCBN/SEBS and AFBS fibers. **e, f** SEM images of the AFBS microfibers coated with Ag NPs

In this research, the BN NPs were functionalized by FC-4430 fluorosurfactant to facilitate their homogeneous dispersion in polymer solution (inset of Fig. 1a). As indicated in Fig. S1, FCBN in trichloromethane/toluene mix solvent showed excellent stability after 20 min storage. Well-dispersed FCBN in SEBS can improve the spinnability of the composite microfibers. As shown in Figs. 1b and Fig. S2a, b, the BN-encapsulated microfibers became more uniform after the addition of fluorosurfactant, and the BN NPs can be observed uniformly distributing inside the SEBS microfibers (Fig. 1c), providing prerequisites for unimpeded heat conduction. It is also notable that the prepared FCBN/SEBS textile showed evenly distributed diameters and favorable porosity for adequate breathability. Besides, the FTIR spectrum of the FCBN/SEBS fibers in Fig. S3b showed two peaks at 1388 cm^−1^ (B-N stretching vibration) and 700 cm^−1^ (B-N formation vibration), confirming that BN NPs were successfully combined with SEBS fibers. Moreover, the characteristic peaks in the XRD patterns (Fig. S3b) also confirm the hybridization of BN NPs (PDF#45–0893) in the SEBS substrates. The abundant incorporation of BN NPs can establish a rapid thermal conduction pathway inside the polymeric fibers (Fig. 1d), thereby providing outstanding thermal comfort for the nonwoven textile. However, due to the presence of numerous thermal insulating interfaces between adjacent fibers, the heat transfer path in the FCBN/SEBS microfibers is restricted solely to the interior of the fibers, hindering the formation of an interwoven three-dimensional heat transfer network.

Ag NPs were grown in situ on the surface of the FCBN/SEBS fibers by hydrazine hydrate reduction of the silver trifluoroacetate (inset of Fig. 1a). As depicted in Figs. 1e, f and Fig. S4, silver nanoparticles are successfully and uniformly loaded onto the surface of nonwoven AFBS textiles without deteriorating the porosity. Observation of the cross section reveals that Ag NPs were evenly distributed on the outer surface of the fibers (Fig. 1f), which can function as a thermally conductive sheath to bridge the insulating interface between adjacent polymer fibers, providing the solution of the last piece of the puzzle in building unimpededly thermal conductive textiles (Fig. 1d). Besides, the XRD pattern of the AFBS textile showed distinct characteristic peaks of Ag at 38.1°, 44.3°, 64.4°, 77.5°, and 81.5° (PDF#04–0783), demonstrating the successful loading of Ag onto the fibers (Fig. S3b). The EDS mapping further demonstrates the uniform coating layer of Ag on the outside of the microfibers, and the successful embedding of BN NPs inside the microfibers (Fig. S5). Finally, highly conductive EGaIn liquid metal was stencil printed on one side of the AFBS textile (inset of Fig. 1a), the obtained UV-EMI shielding and thermally conductive e-textile can be employed for human health monitoring under extreme conditions, such as high temperatures, electromagnetic radiation, and ultraviolet radiation.

**Fig. 2:**
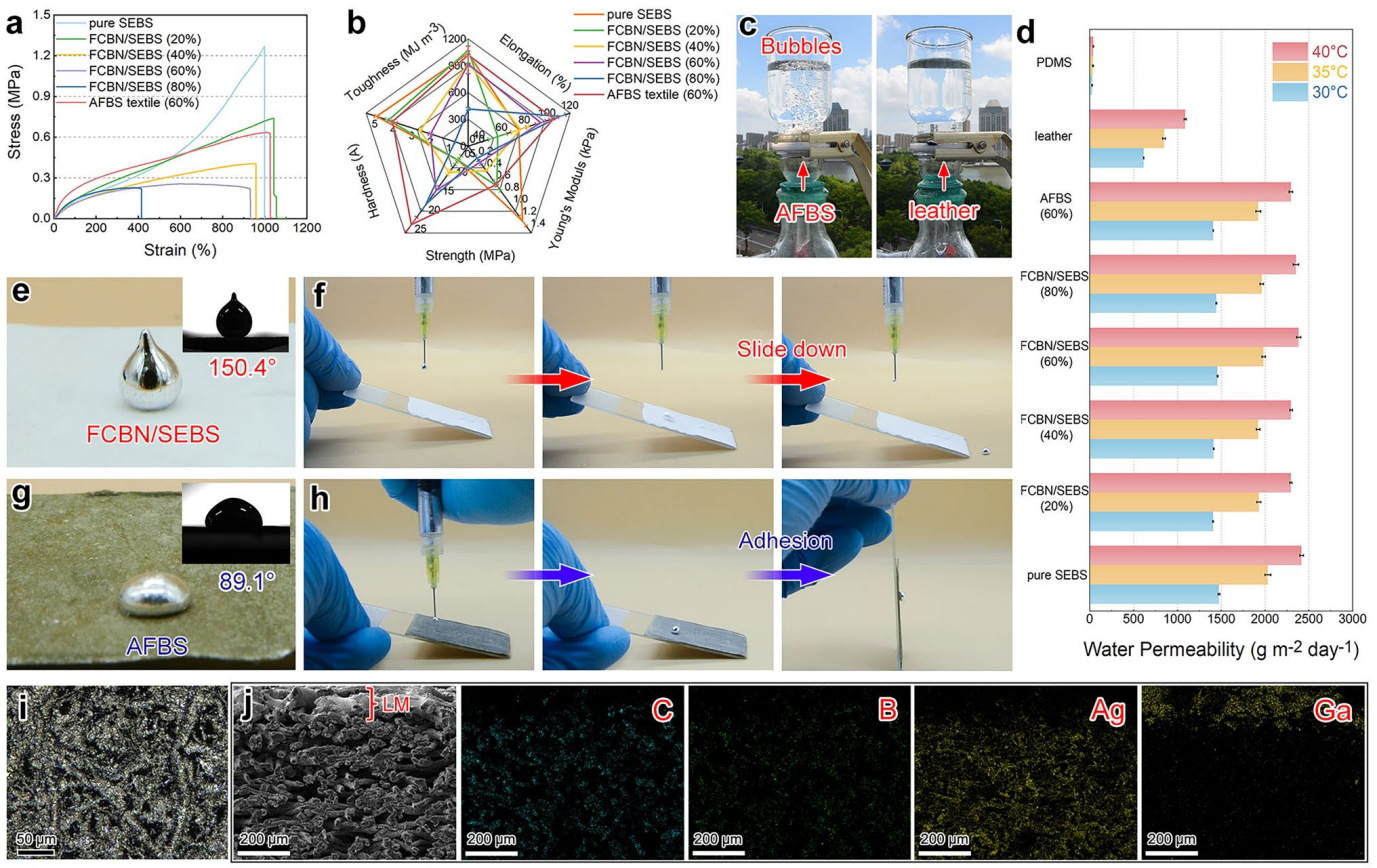
**a** Strain–stress curves and **b** corresponding mechanical properties of different samples. **c** Demonstration on the air permeability of the AFBS textile (versus leather, 500 μm thickness for both samples). **d** Water permeability of different samples. **e, f** Contact angle and surface adhesion of EGaIn droplets on the FCBN/SEBS textile. **g, h** Contact angle and surface adhesion of EGaIn droplets on the AFBS textile. **i** Microscopic observation on the surface morphology and **j** cross section of EGaIn-printed AFBS textile, and the corresponding elemental mapping

Owning to the uniform distribution of BN NPs in the microfibers, both FCBN/SEBS and AFBS textiles exhibited excellent mechanical properties. As shown in Fig. 2a, b, the stress of the elastomeric textiles decreases with increasing the load and breaks at the maximum strain. Amongst, AFBS (60 wt% BN NPs) showed the best overall mechanical properties, particularly Young’s modulus and hardness. Specifically, the elongation, Young’s Modules, strength, hardness, and toughness of AFBS textile was 1020.1 ± 99.3%, 105.5 ± 3.8 kPa, 0.6 ± 0.1 MPa, 25.7 ± 1.8 A, 4.1 ± 0.2 MJ m^−3^, respectively (Fig. 2b), meeting the acquirements for nearly all wearing scenarios. In addition to mechanical properties, air/moisture permeability is important for wearable electronics to enable prolonged use without irritating the skin. As demonstrated in Fig. 2c, the AFBS textile was used as a filter paper, below is a flask connecting to a pump and the upper flask was filled with water to assess its permeability by observing the air bubbles. A large number of air bubbles appeared in the upper flask above the AFBS textile, while a small number of bubbles emerged in the relatively less breathable leather, confirming that the AFBS textile has a favorable air permeability. Moreover, the specific moisture permeability of different textiles was further determined using the weight loss method at temperatures similar to the human skin (30, 35, and 40 °C, 80 RH%) (Fig. 2d). Specifically, pure SEBS (2030.9 g m^−2^ day^−1^), FCBN/SEBS (1919.9–1982.3 g m^−2^ day^−1^) and AFBS (1921.6 g m^−2^ day^−1^) exhibited far higher moisture permeability than leather (847.7 g m^−2^ day^−1^) and casted PDMS film (3.2 g m^−2^ day^−1^) under 35 °C.

Regarding flexible conducting materials, liquid metal exhibits exceptional characteristics such as ultra-high conductivity, extreme ductility, and superior biocompatibility. However, it is noteworthy that liquid metal possesses a significantly high surface tension and exhibits low affinity for SEBS substrates. As demonstrated in Fig. 2e, the contact angle (CA) of LM on FCBN/SEBS textile was found to be 150.4°, indicating the poor surface affinity of the LM. As a result, the LM droplet positioned on the FCBN/SEBS textile slipped off upon contacting the textile surface (Fig. 2f and Video S1). Interestingly, the CA of liquid metal on AFBS textile greatly decreased to 89.1° due to the fast alloying between silver and EGaIn [50], demonstrating the LM hydrophilicity of the Ag NPs-plated microfibers (Fig. 2g). As depicted in Fig. 2h and Video S2, the LM showed good affinity to the AFBS textile, and therefore, it can steadily adhere to the substrates regardless of the tilting angle. Benefiting from the significantly improved affinity of LM to the textile substrate, stencil printing and pre-stretching strategies were employed to form a stable and conductive LM circuit on AFBS textiles [51]. SEM observation and EDS mapping on the cross section of LM-printed e-textile show that the EGaIn was tightly coated on the surface of every AFBS fiber (Fig. 2i, j), providing the functionality of health monitoring while maintaining the breathability of the nonwoven substrates. The conductivity tests under tensile reveal that the electrical resistance of the electronic textile is only 0.9 Ω at an 800% strain (Fig. S6), indicating minimal variation compared to its initial state.

### UV-EMI Shielding Performance of the E-Textile

The deleterious impact of ultraviolet (UV) radiation on human health is well documented in the literature. Prolonged exposure to UV radiation poses a significant risk to the skin, manifesting in adverse outcomes such as DNA damage, ultimately elevating the susceptibility to skin cancers [52]. UVA at wavelengths of 315–400 nm accelerates skin aging and cancer, and UVB at wavelengths of 280–315 nm may cause inflammation and redness of the skin [53]. Common UV-proof materials such as TiO_2_ are effective in absorbing UV radiation; however, their photocatalytic properties cause the production of oxygen radicals, which may damage the structure of the polymer matrix/substrate. Fortunately, the resonance frequency of free electrons in Ag NPs matches the frequency of the incident UV light, which produces a strong extinction effect (localized surface plasmon resonance (LSPR)), and this portion of photon energy is either absorbed or scattered by the Ag NPs, which ultimately release in the form of heat, rendering Ag NPs with excellent UV shielding properties [54].

**Fig. 3:**
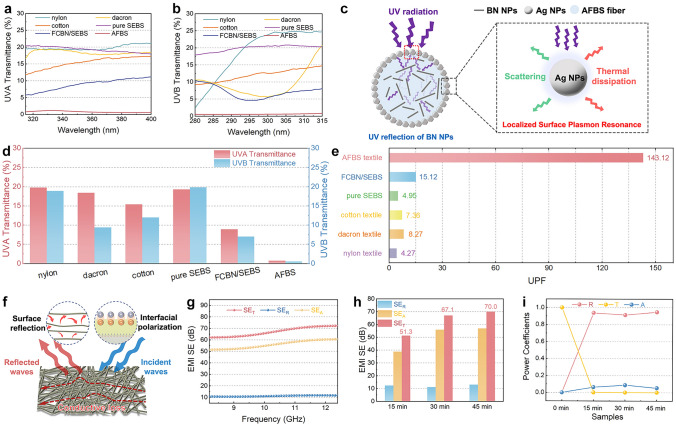
**a** UVA and **b** UVB transmittance of different textiles. **c** UV shielding mechanism of BN NPs and Ag NPs in/on AFBS microfibers. **d** Average UVA and UVB transmittance, and **e** UPF values of different samples. **f** EMI shielding mechanism of AFBS textiles. **g** Electromagnetic shielding effectiveness of the AFBS textile after 30 min Ag plating. **h** SE_*T*_, SE_*A*_ and SE_*R*_ values, and **i** power coefficients of AFBS textiles with different Ag plating times

As displayed in Fig. S7a–c, Ag NPs exhibited excellent UV absorption (58.2%) and reflectivity (20.9%) properties compared to conventional TiO_2_ NPs (23.5% and 51.5%), thus ultimately manifested superior UV shielding properties (transmittance = 20.8%) compared to TiO_2_ NPs (transmittance = 25.0%). Unexpectedly, BN NPs also showed good UV reflectivity (72.7%), which may further enhance the UV-protecting properties of the nonwoven textiles. As shown in Fig. 3a, b, commercial textiles (nylon textile, Dacron textile, and cotton textile) exhibit high UV transmittance, thus a large number of UV radiation can pass through the textiles. Fortunately, AFBS textiles are able to effectively shield both UVA and UVB radiations, providing excellent UV-proof capabilities for skin health. As a result of the uniformly loaded Ag NPs on the surface of the elastomeric matrix, the AFBS microfibers can absorb UV energy and release it in the form of heat (LSPR effect) when exposed to UV radiation. In addition, the BN NPs encapsulated inside the microfibers have prominent reflectivity against UV; hence, the penetrated UV waves can be efficiently reflected and absorbed by the Ag NPs. Therefore, the combination of two nanoparticles endows the AFBS textile with unparalleled UV protection performance (Fig. 3c). The ultraviolet protection factor (UPF) is a quantitative measurement employed to assess the efficacy of a material or fabric in attenuating UV radiation penetration. Materials with UPF ≥ 50 are considered to have UV protective performance. Here, the UPF values can be calculated by the following equations (European Standard EN 13758–2):

1 $$T\left( {{\text{UVA}}} \right)_{i} = \frac{1}{m}\mathop \sum \limits_{\lambda = 315}^{\lambda = 400} T_{i} \left( \lambda \right)$$

2 $$T\left( {{\text{UVB}}} \right)_{i} = \frac{1}{k}\mathop \sum \limits_{\lambda = 290}^{\lambda = 315} T_{i} \left( \lambda \right)$$

where “$T\left( {{\text{UVA}}} \right)_{i}$” and “$T\left( {{\text{UVB}}} \right)_{i}$” are the arithmetic means of each sample, “*k*” and “*m*” represent the testing times, and “$T_{i} \left( \lambda \right)$” is the transmittance of sample “*i*” at wavelength “*λ*”.

3 $${\text{UPF}} = \frac{{\mathop \sum \nolimits_{\lambda = 280}^{\lambda = 400} \;E\left( \lambda \right) \cdot \varepsilon \left( \lambda \right) \cdot \Delta \left( \lambda \right)}}{{\mathop \sum \nolimits_{\lambda = 280}^{\lambda = 400} \;E\left( \lambda \right) \cdot T\left( \lambda \right) \cdot \varepsilon \left( \lambda \right) \cdot \Delta \left( \lambda \right)}}$$

where “$E\left( \lambda \right)$” is the solar spectral irradiance (W m^−2^ nm^−1^), “$\varepsilon \left( \lambda \right)$” is the relative erythema effectiveness, “$\Delta \left( \lambda \right)$” is the wavelength interval of measurements (nm), and “$T\left( \lambda \right)$” is the spectral transmittance at wavelength *λ*.

As presented in Fig. 3d, e, conventional textiles have high UV transmittance and poor UPF performance, e.g., cotton textile shows UVA and UVB transmittance of 15.4% and 12.1%, respectively, with a UPF of 7.3. Pure SEBS nonwoven microfibers have UVA and UVB transmittance of 19.3% and 19.9%, respectively, yielding a UPF of 4.9. In contrast, the transmission of UVA and UVB through the FCBN/SEBS textiles embedded with BN NPs drastically decreased to 8.9% and 7.0%, respectively, which was attributed to the reflective properties of BN NPs against UV. More excitingly, AFBS textiles with Ag NPs coating manifest a much higher UVA and UVB transmission shielding performance of 0.7% and 0.5%, respectively, delivering the highest UPF value of 143.12. This performance also surpasses some recently reported UV protective textiles (Table S1). Therefore, the excellent UV protective performance of the AFBS textile reduces the potential for adverse effects such as sunburn and long-term skin damage during outdoor activities.

Moreover, electromagnetic pollution originating from electronics, power lines, and radiofrequency devices not only affects the sensing and transmitting performance of devices, but also endangers the physiological health of humans [55]. Therefore, advanced skin-attachable electronics are craving for EMI shielding functions. In this work, the AFBS nonwoven textiles are uniformly coated with Ag NPs, forming excellent conductive networks with microporous structures. When EMW are irradiated onto the surface of the highly conductive microfibers, an impedance mismatch occurs, leading to a substantial number of reflections of EMW reflections. On the other hand, the EMW gets through the AFBS textile interacts with the mobile charge carriers in the conductive network of the AFBS textile and generates an induced current, thus realizing the conductive loss of EMW. In addition, the EMW entering the interior of the AFBS textile undergoes multiple internal reflections during its passage through the micro pores of the conductive textile, resulting in multiple attenuation of the EMW until the energy is completely absorbed (Fig. 3f). Figures 3g, h and S8 indicate that the AFBS textile has excellent shielding performance in the X-band (8.2–12.4 GHz) after 15, 30, and 45 min of Ag- plating treatment, achieving shielding effectiveness (SE_*T*_) values of 51.3, 67.1, and 70.0 dB, respectively. It is clear that this EMI shielding performance far exceeds the threshold for commercial applications (> 20 dB) when the treatment time is beyond 15 min, and is also superior to those of recently reported EMI shielding textiles (Table S2). Moreover, three power coefficients (absorption (*A*), reflection (*R*), and transmission (*T*)) were obtained from the measured scattering parameters to further explore the main shielding mechanisms. As indicated in Fig. 3i, when the treatment time is longer than 15 min, the *R*-value surges to 0.9 and remains stable, implying that EMW reflectivity is the primary shielding mechanism. When the incident EMW encounters the AFBS textile, an impedance mismatch results in the surface reflection of a majority of EMW, dissipating it by transforming the electromagnetic energy into alternative forms, such as heat.

### Unimpeded Thermal Dissipation Performance of the AFBS Textiles

**Fig. 4:**
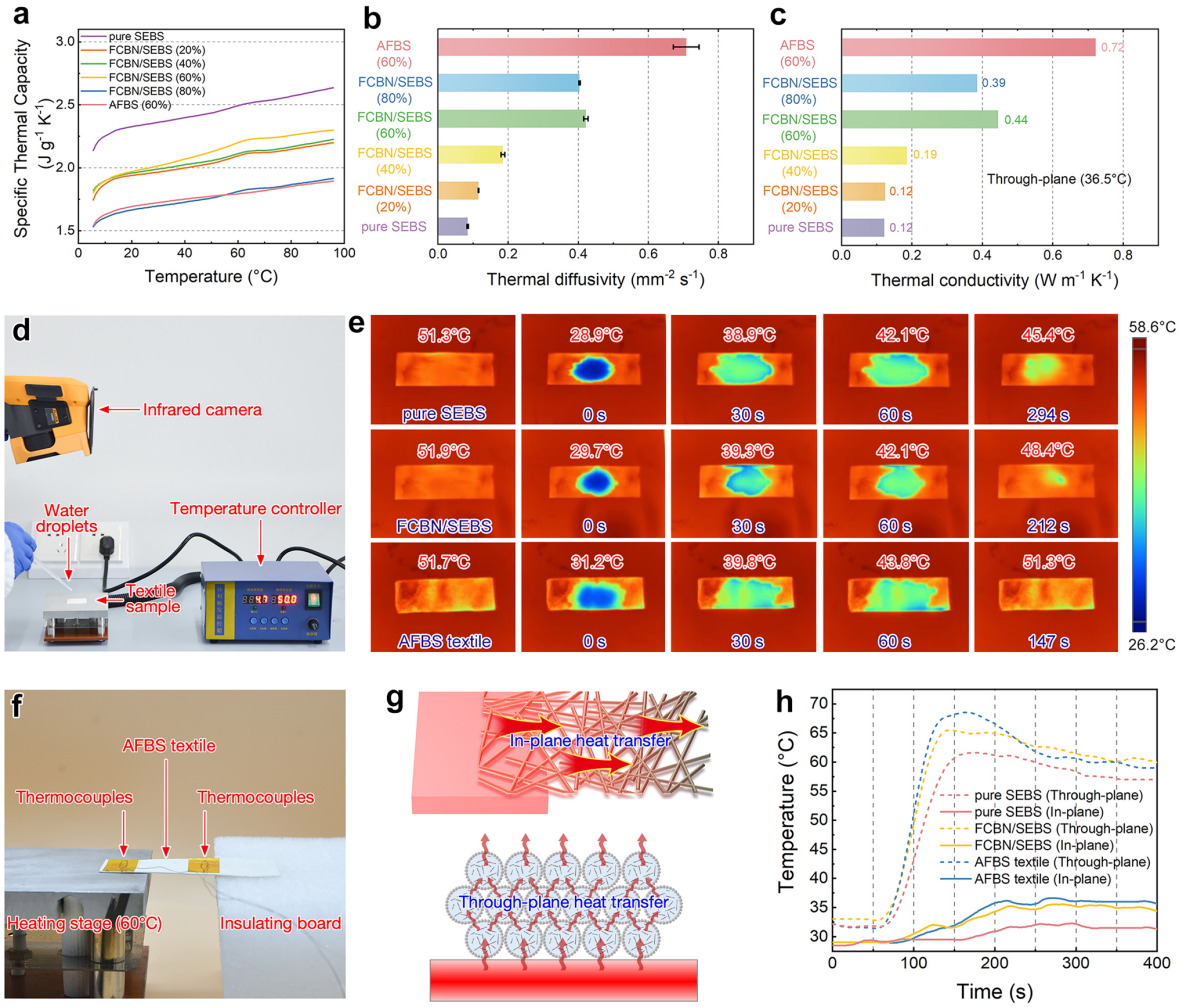
**a** Specific thermal capacity of pure SEBS, FCBN/SEBS (with various BN loading) and AFBS (60 wt% BN loading) textiles. Through-plane **b** thermal diffusivity and **c** thermal conductivity of different samples. **d** Setup for the water evaporation rate tests. **e** Infrared thermal images of water droplets on pure SEBS textile, FCBN/SEBS textile (60 wt% BN loading) and AFBS textile placed on a 55 °C heating stage. **f** A homemade setup and **g** a schematic for the practical in-plane and through-plane thermal conductivity tests, **h** and corresponding temperature changes of different samples during the tests

Skin-attachable electronics need accessibility to heat dissipation to maintain thermal comfort on the human skin [56, 57]. As illustrated in Fig. 1d, BN NPs with excellent thermal conductivity are uniformly connected and embedded inside the AFBS microfibers to realize efficient phonon transmission and low contact thermal resistance. In addition, the uniformly plated Ag NPs on the microfiber further improve the heat transfer performance via bridging the insulating interface between adjacent polymer fibers. Two factors together lead to a highly efficient thermal conductivity network for unimpeded heat dissipation. The differential scanning calorimetry (DSC) sapphire analysis was first used to test the heat release/absorption capacity of different textiles under 5–95 °C temperature changes. In order to better fit the temperature of human skin, the specific thermal capacities corresponding to the respective samples at 36.5 °C were used for the subsequent research and calculation (Fig. 4a). As depicted in Fig. 4b, the thermal diffusion coefficient exhibits an upward trend with the escalation of BN NPs loading. At 60 wt%, the interconnected BN NPs establish a robust thermal conductivity network along the interconnected fibers. However, exceeding 60 wt% BN NPs loading induces brittleness in the FCBN/SEBS fibers, consequently compromising their mechanical properties. Consequently, a BN NPs loading of 60 wt% was chosen for subsequent investigations. As expected, Ag NPs on the surface of the AFBS microfibers further improved the thermal diffusion properties to 69.0%. Further, the specific thermal conductivity can be calculated through the following equation:

4 $$\alpha = \frac{k}{\rho c}$$

where *k* is the thermal conductivity (W m^−1^ K^−1^), $\alpha$ is the thermal diffusion coefficient (m^2^ s^−1^), $\rho$ is the density (kg m^−3^), and *c* is the specific heat capacity measured by the sapphire method (J kg^−1^ K^−1^). The thermal conductivities of various samples are illustrated in Fig. 4c. It is evident that the conductivity noticeably decreased (0.39 W m^−1^ K^−1^) upon reaching an 80% increase in the BN NPs content. Nevertheless, the thermal conductivity of AFBS experienced a further enhancement, reaching 0.72 W m^−1^ K^−1^ after coating the microfibers with Ag NPs, far exceeding those of previously reported textiles (Table S3). This underscores the crucial role of Ag coating in augmenting interfacial heat conductivity.

To further investigate the unobstructed heat dissipation properties of the elastomeric nonwoven textiles, pure SEBS textile, FCBN/SEBS textile (60 wt% BN loading), and AFBS textile with the same thickness of 200 μm were heated on a thermostatic heating platform at 55 °C. 50 μL water was dropped on the surface of each textile, and the times taken for the complete water evaporation were recorded using an infrared camera (Fig. 4d). As shown in Fig. 4e, pure SEBS textile took over 294 s to evaporate due to the low thermal conducting performance of the polymer substrate, making it difficult for heat to be transferred from the heating stage to the upper surface of the textile. FCBN/SEBS textile took 212 s for the complete evaporation because the BN NPs inside the SEBS fibers can accelerate the diffusion of heat and ultimately accelerate the vaporization. In sharp contrast, water droplets on the AFBS textile evaporated within a remarkably short time of 147 s. This rapid evaporation can be attributed to the AFBS textile’s exceptional unimpeded heat transfer properties, facilitating efficient heat conduction to the wetted area and accelerating water evaporation. Lastly, the through-plane and in-plane thermal conductive performance of the AFBS nonwoven textile was evaluated in a homemade setup. As demonstrated in Fig. 4f, g, one end of a textile sample in 5 cm × 1 cm × 300 μm was attached to a thermostatic heating stage (60 °C), while the other end was positioned on an insulating foam board. The through-plane and in-plane temperatures were recorded by K-type thermocouples, which were directly attached to the upper surface of both ends. The heat transfer results in Fig. 4h indicate that AFBS exhibits a faster temperature rise in both directions. Specifically, the in-plane heat transfer speed and maximum temperature are significantly lower than those in through-plane direction, a consequence of the longer heat transfer distance within the textile. This outcome further highlights the unimpeded heat transfer pathways within AFBS composite fibers.

### E-Textiles for Epidermal Electrophysiological Monitoring

Thanks to the rational integration of thermal conductivity and UV-EMI shielding functionalities, the AFBS-based e-textile printed with extremely conductive LM not only provides thermal comfort and health protection performance, but also can be utilized as skin-attachable bio-electrodes for health monitoring. Physiological signals such as sEMG, ECG, and EEG are derived from the weak activities of the human body, which are susceptible to external electromagnetic interferences. Fortunately, the fabricated e-textiles possess electromagnetic shielding properties that isolate the EMW from physiological acquisition, enabling high-fidelity monitoring of various health indicators.

**Fig. 5:**
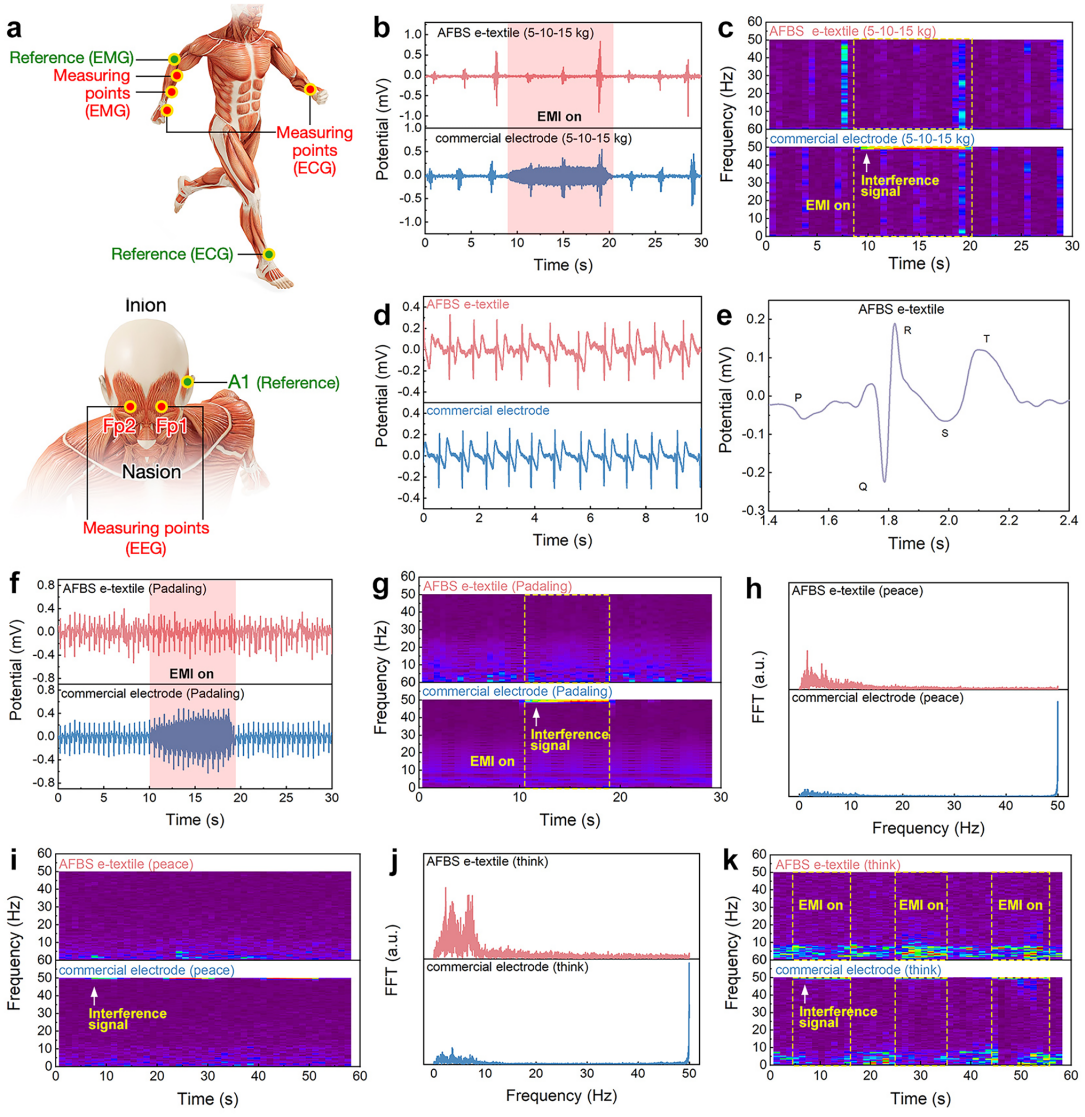
**a** Electrode position for electrophysiological monitoring. **b, c** EMG signals recorded by commercial electrodes and AFBS e-textiles, tests were carried out under varying gripping motions and strong electromagnetic interference (indicated by pink area). **d, e** ECG signals acquired by the commercial electrodes and AFBS e-textiles. **f, g** ECG signals correspond to moderate exercise (pedaling) under strong electromagnetic interference. EEG signals monitored by commercial electrodes and AFBS e-textiles during **h, i** peace state and **j, k** thinking state under normal conditions and strong electromagnetic interference

Figure 5a illustrates the positions of the e-textile bioelectrodes for monitoring sEMG, ECG, and EEG signals. sEMG is of substantial interest in the diagnosis of muscle diseases and musculoskeletal disorders. As shown in Fig. 5b, c, the AFBS e-textiles and commercial electrodes were used to monitor the sEMG signals of human muscle, when applying a gradually increased gripping force of 5–15 kg with enhanced electromagnetic interference (3 A and 50 Hz AC generated by an electric fan, Fig. S9). It can be seen that the AFBS e-textile can reproducibly detect sEMG signals at different grip forces with stability comparable to that of commercial electrodes. In addition, the AFBS e-textile also demonstrates excellent immunity to electromagnetic interference when compared to commercial electrodes under enhanced electromagnetic interference (50 Hz for 10 s, Video S3). Contrarily, the sEMG signals of the commercial electrodes were severely distorted when subjected to strong EMI, where obvious noise at 50 Hz can be seen in the frequency domain plot (Fig. 5c, Video S4). Furthermore, ECG signals at resting and exercising (e.g., pedaling) situations were also monitored using both bioelectrodes. It was evident that the ECG signals detected by the AFBS e-textile were highly stable and reproducible, comparable to commercial electrodes (Fig. 5d). The amplified ECG pattern detected by the AFBS bioelectrodes can clearly reveal the P, QRS, and T peaks, which are important indicators of the physiological signals of the human heart (atrial depolarization, left and right ventricular depolarization and ventricular repolarization) (Fig. 5e). Figure 5f shows that when exposed to strong electromagnetic interference, the ECG signals acquired by the commercial electrodes are dominated by serious noise in the frequency-domain plot (Fig. 5g). In comparison, AFBS bioelectrodes were able to record ECG signals clearly and accurately without much noise affecting the normal patterns (as highlighted in Fig. 5g).

Human EEG signals can be used for the monitoring of brain activities in different states, which can be divided into five frequency bands (i.e., *δ* wave (0–4 Hz), θ wave (4–8 Hz), *α* wave (8–12 Hz.), *β* wave (12–40 Hz), and γ wave (> 40 Hz)). In this case, the *δ* wave indicates that the brain is in a resting state, while stronger signals in the high-frequency bands (θ wave, *α* wave, and *β* wave) imply that the human brain is in a highly aroused state of mental concentration. As shown in Fig. 5h, i, the AFBS e-textile and commercial electrodes were both employed to monitor the EEG signals under the interference of electromagnetic waves. A large number of alpha waves (0–4 Hz) could be seen in Fig. 5h, corresponding to the inactive state of the brain. However, the EEG signal captured by the commercial electrodes suffered from significant interference of electromagnetic sources, and noticeable noise signals severely affected the clarity of the *α* wave (~ 50 Hz). The emergence of signals in 50 Hz in the frequency diagram further confirms that commercial electrodes lack enough immunity to EMI (Fig. 5i). Furthermore, EEG signals in the high-frequency cerebral activities (mathematical calculation, > 4 Hz) were also captured by the AFBS e-textile, which also can be recorded accurately without much noise compared to commercial electrodes (Fig. 5j, k). The above results prove that our UV-EMI shielding AFBS e-textile succeeded in monitoring high-quality sEMG, ECG and EEG signals in complex electromagnetic environments.

## Conclusions

In summary, this study has successfully developed an electrophysiological monitoring e-textile that prioritizes wearer comfort through enhanced air permeability and heat dissipation, while concurrently providing health-protective features such as UV-EMI resistance. The achievement of these properties is attributed to the integrated thermal conducting fiber network with seamless thermal interfaces constructed by BN NPs encapsulation and Ag NPs plating. The resulting AFBS textile exhibits notable characteristics, including a high thermal conductivity facilitating unimpeded thermal dissipation (0.72 W m ^−1^ K^−1^) and exceptional moisture permeability (2294.8 ± 20.4 g m^−2^ day^−1^). Remarkably, the uniform loading of Ag NPs on elastic microfibers imparts excellent UV protection (UPF = 143.1) and electromagnetic shielding performance (SE_*T*_ > 65, X-band) to the AFBS textile, enhancing its health-protective capabilities. Moreover, through the homogeneous printing of LM, the AFBS e-textile serves as an EMI-resistant epidermal electrode. Consequently, the UV-EMI shielding AFBS e-textile, when exposed to severe electromagnetic interference, enables the high-fidelity detection of human physiological signals (sEMG, ECG, and EEG).

## Supplementary Information

Below is the link to the electronic supplementary material.

Supplementary file1 (PDF 795 KB)

Supplementary file2 (MP4 1885 KB)

Supplementary file3 (MP4 2716 KB)

Supplementary file4 (MP4 2040 KB)

Supplementary file5 (MP4 1974 KB)
