# Supplementary material for: Thermally Conductive and UV-EMI Shielding Electronic Textiles for Unrestricted and Multifaceted Health Monitoring
Source: Nanomicro Lett. 2024 May 21;16:199. doi: 10.1007/s40820-024-01429-x (PMC11109083; doi:10.1007/s40820-024-01429-x)
Supplement: Supplementary file 1 — Supplementary file1 (PDF 795 KB) [file 40820_2024_1429_MOESM1_ESM.pdf]

Supporting Information for

Thermally Conductive and UV-EMI Shielding Electronic Textiles for Unrestricted and Multifaceted Health Monitoring

Yidong Peng[1], Jiancheng Dong[1], Jiayan Long[1], Yuxi Zhang[1], Xinwei Tang[1], Xi Lin[1], Haoran Liu[1], Tuoqi Liu[1], Wei Fan[1], Tianxi Liu[1,*], Yunpeng Huang[1,*]

[1] Key Laboratory of Synthetic and Biological Colloids, Ministry of Education, School of Chemical and Material Engineering, Jiangnan University, Wuxi 214122, P. R. China

*Corresponding authors. E-mail: txliu@jiangnan.edu.cn (Tianxi Liu), hypjnu@jiangnan.edu.cn (Yunpeng Huang)

Supplementary Figures and Tables

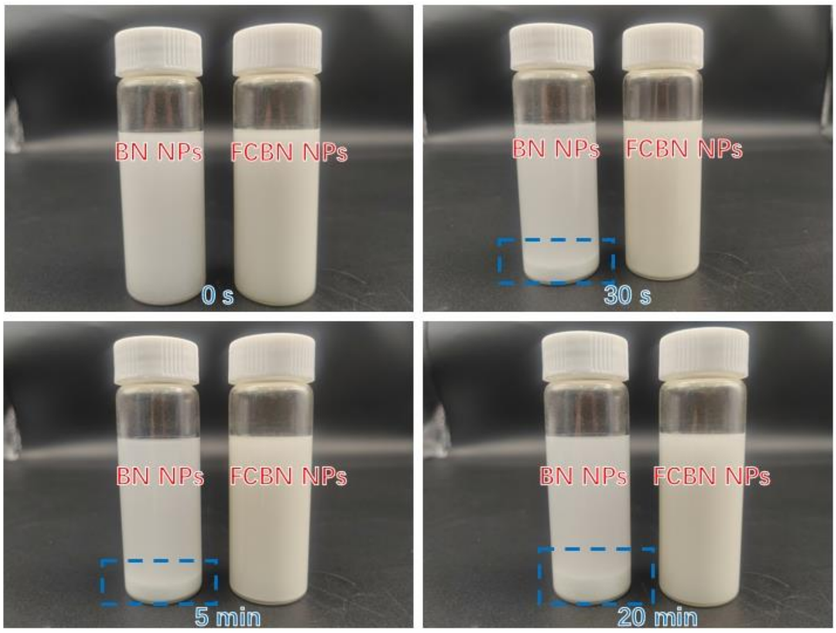

Fig. S1 Photographs of BN NPs and FCBN NPs dispersion (in trichloromethane/toluene solvent) stored for 30 s, 5 min and 20 min, respectively

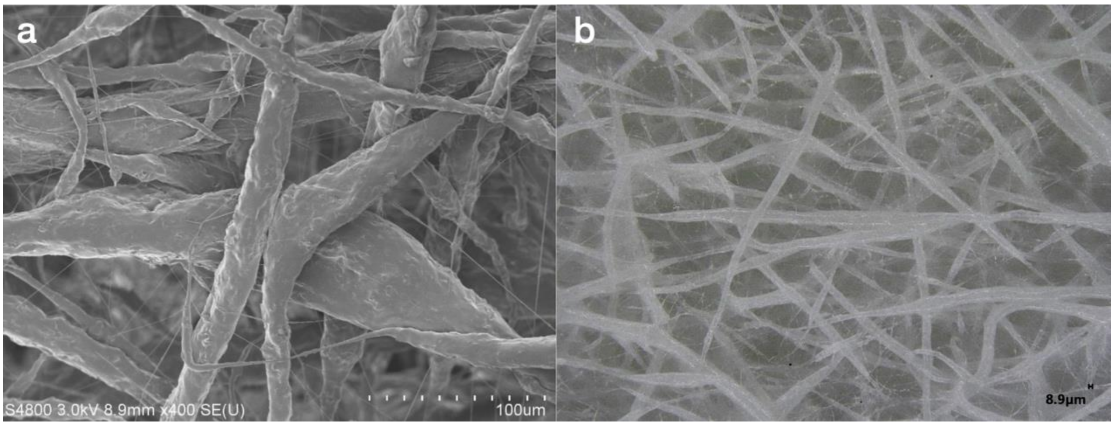

Fig. S2 (**a**) SEM image of BN-encapsulated SEBS microfibers without the addition of FC-4430. (**b**) A low magnification SEM image of FCBN/SEBS microfibers

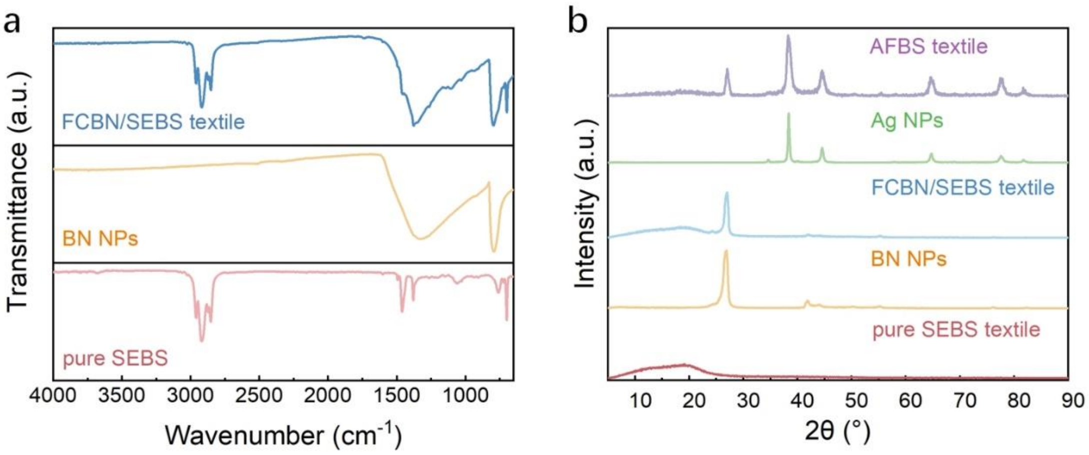

Fig. S3 (**a**) FT-IR spectra of pure SEBS, BN NPs and FCBN/SEBS. (**b**) XRD patterns of pure SEBS, BN NPs, FCBN/SEBS, Ag NPs and AFBS

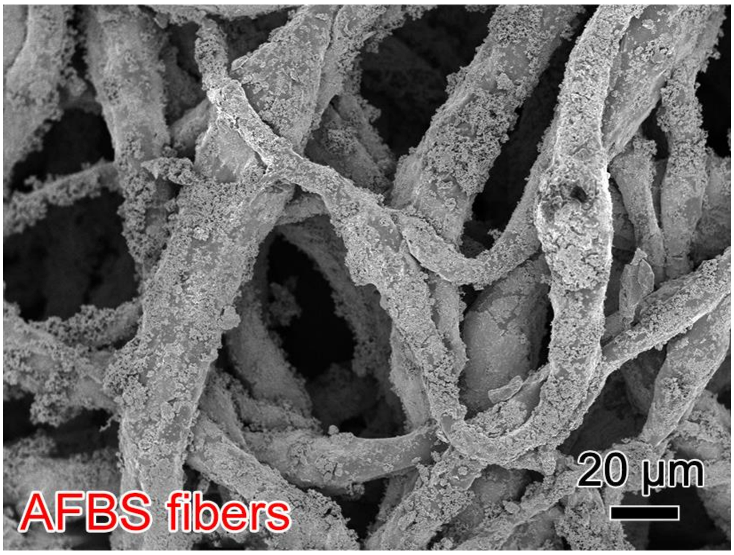

Fig. S4 SEM image of AFBS microfibers at low magnification

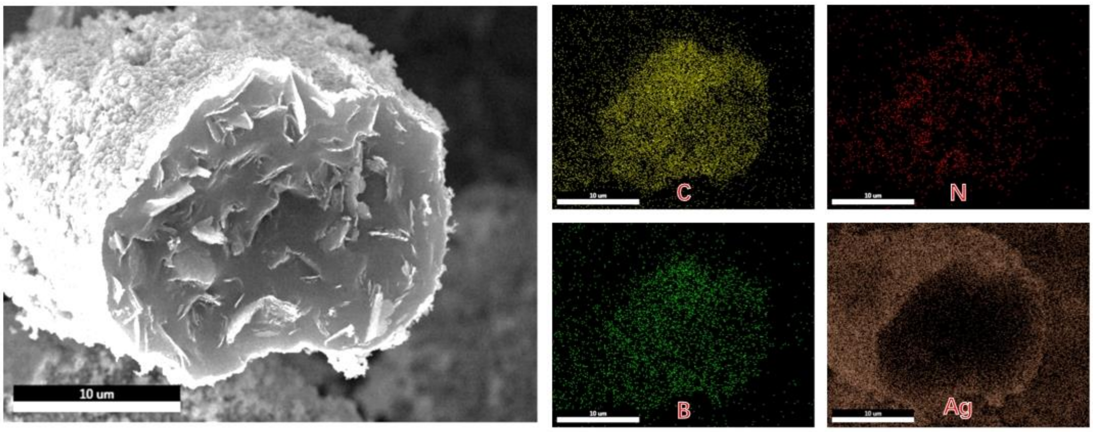

Fig. S5 EDS mapping on the cross-section of AFBS fiber, showing the distribution of the elements C, N, B, and Ag

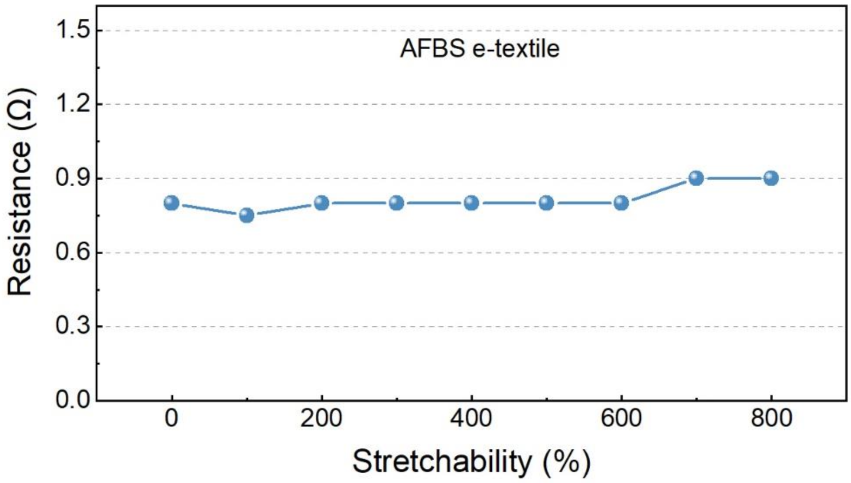

Fig. S6 The resistance change of the AFBS e-textile when stretched up to 800%

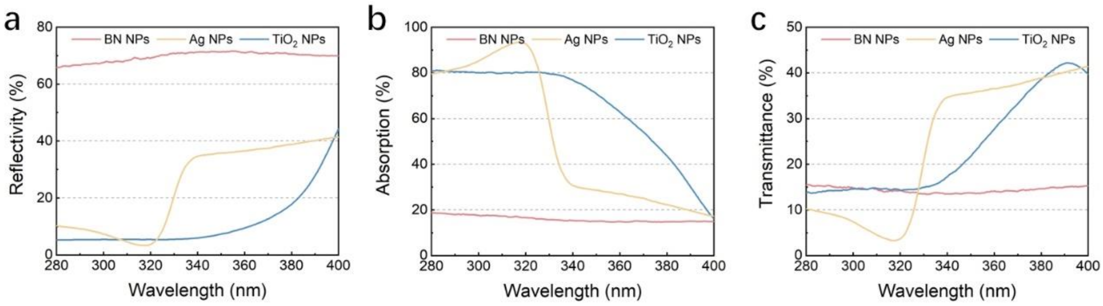

Fig. S7 (**a**) Reflectivity, (**b**) absorption and (**c**) transmittance of BN NPs, Ag NPs and TiO_2 NPs in the UV band

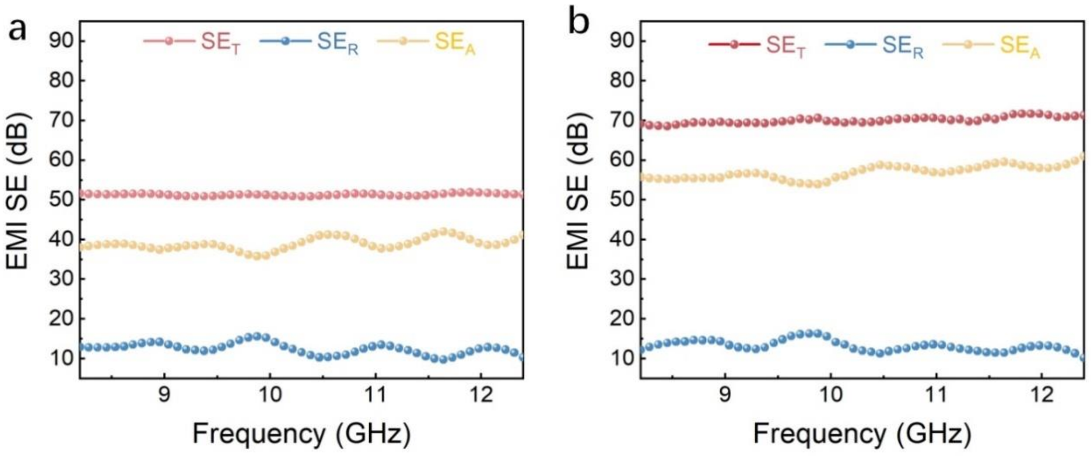

Fig. S8 Electromagnetic shielding effectiveness of the AFBS e-textiles after silver plating treatment for (**a**) 15 min and (**b**) 45 min

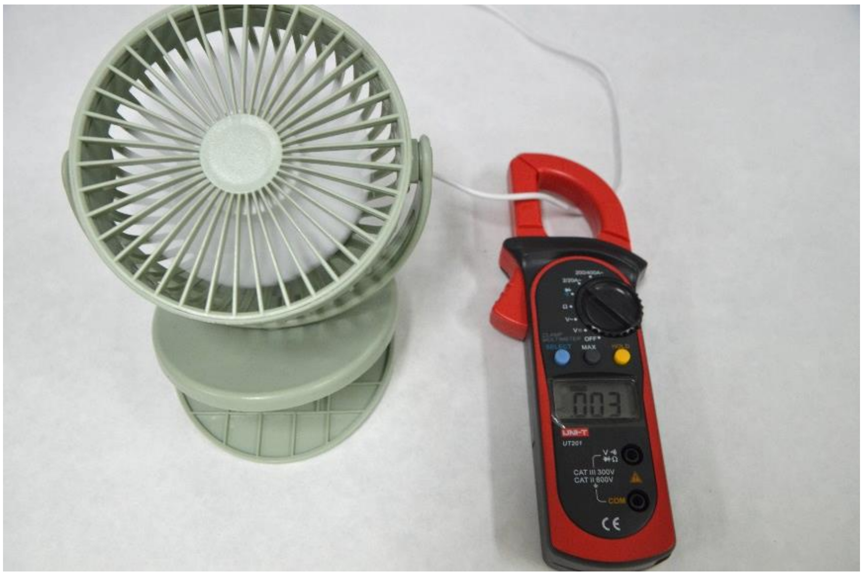

Fig. S9 Alternating current of 3A and 50 Hz AC generated by a fan to simulate the external electromagnetic interference

Table S1 Comparison of the UV protection performance between AFBS e-textile and previously reported textiles

Samples	UPF	References
PI-cotton textile	34.9	[S1]
Ag-doped TiO_2 hybrid textile	41.0	[S2]
TiO_2@ployester cotton	55.8	[S3]
ZnO/SiO_2@cotton textile	123.9	[S4]
CeO_x@HNTs anti-biofilm	58.5	[S5]
**AFBS e-textile**	**143.1**	**This work**

Table S2 Comparison of the EMI (X-band) performance between AFBS e-textile and previously reported textiles

Samples	EMI shielding effectiveness	References
AgNW@cotton textile	38.2	[S6]
MXene@GA-CF textile	35.0	[S7]
Pt/PDA@PI textile	53.0	[S8]
AgNW/MXene/TPU	41.7	[S9]
**AFBS e-textile**	**67.1**	**This work**

Table S3 Comparison of the thermal conductivity performance between AFBS e-textile and previously reported textiles

Samples	Thermal conductivity	References
FP-Cool (BN) textile	0.14	[S10]
BNNS@TPU textile	0.18	[S11]
BN@Flax fiber textile	0.36	[S12]
BNNS@TPU@Cellulose textile	0.31	[S13]
**AFBS e-textile**	**0.72**	**This work**

Supplementary References

[S1] D. Song, B. Jiang, J. Zhou, Y. Ouyang, Y. Zhang et al., Soluble and colorless polyimide coated cotton fabric with attractive multifunction: Warmth retention, breathable, antifouling, UV and acid resistance. Chem. Eng. J. **455**, 140755 (2023). https://doi.org/10.1016/j.cej.2022.140755

[S2] M. Mamunur Rashid, B. Tomšič, B. Simončič, I. Jerman, D. Štular et al., Sustainable and cost-effective functionalization of textile surfaces with Ag-doped TiO_2/polysiloxane hybrid nanocomposite for UV protection, antibacterial and self-cleaning properties. Appl. Surf. Sci. **595**, 153521 (2022). https://doi.org/10.1016/j.apsusc.2022.153521

[S3] H. Rabiei, S. Farhang Dehghan, M. Montazer, S.S. Khaloo, A.G. Koozekonan UV protection properties of workwear fabrics coated with TiO_2 nanoparticles. Front. Public Health **10**, 929095 (2022). https://doi.org/10.3389/fpubh.2022.929095

[S4] M.M. Abd El-Hady, S. Sharaf, A. Farouk Highly hydrophobic and UV protective properties of cotton fabric using layer by layer self-assembly technique. Cellulose **27**, 1099–1110 (2020). https://doi.org/10.1007/s10570-019-02815-0

[S5] Y. Feng, D. Zhang, X. Chen, C. Zhou, M. Liu Confined-synthesis of ceria in tubular nanoclays for UV protection and anti-biofilm application. Adv. Funct. Mater. **34**, 2307157 (2024). https://doi.org/10.1002/adfm.202307157

[S6] L. Xing, H. Yang, X. Chen, Y. Wang, D. Sha et al., Caffeic acid induced *in situ* growth of AgNWs on cotton fabric for temperature and pressure sensing and electrical interference shielding. Chem. Eng. J. **471**, 144620 (2023). https://doi.org/10.1016/j.cej.2023.144620

[S7] B. Yan, X. Bao, Y. Gao, M. Zhou, Y. Yu et al., Antioxidative MXene@GA-decorated textile assisted by metal ion for efficient electromagnetic interference shielding, dual-driven heating, and infrared thermal camouflage. Adv. Fiber Mater. **5**, 2080–2098 (2023). https://doi.org/10.1007/s42765-023-00330-3

[S8] X. Tang, X. Zhao, Y. Lu, S. Li, Z. Zhang et al., Flexible metalized polyimide nonwoven fabrics for efficient electromagnetic interference shielding. Chem. Eng. J. **480**, 148000 (2024). https://doi.org/10.1016/j.cej.2023.148000

[S9] J. Dong, Y. Feng, K. Lin, B. Zhou, F. Su et al., A stretchable electromagnetic interference shielding fabric with dual-mode passive personal thermal management. Adv. Funct. Mater. **34**, 2310774 (2024). https://doi.org/10.1002/adfm.202310774

[S10] F. Li, S. Wang, Z. Wang, K. Jiang, X. Zhao et al., Fouling-proof cooling (FP-cool) fabric hybrid with enhanced sweat-elimination and heat-dissipation for personal thermal regulation. Adv. Funct. Mater. **33**, 2370020 (2023). https://doi.org/10.1002/adfm.202370020

[S11] D. Miao, X. Wang, J. Yu, B. Ding A biomimetic transpiration textile for highly efficient personal drying and cooling. Adv. Funct. Mater. **31**, 2008705 (2021). https://doi.org/10.1002/adfm.202008705

[S12] Y. Lu, Z. Li, N. Guo, P. Zhang, S. Fu Preparation and properties of boron nitride loaded regenerated cooling fiber. Fibres. Polym. **24**, 1633–1640 (2023). https://doi.org/10.1007/s12221-023-00084-0

[S13] X. Huang, Z. Li, Y. Li, X. Wu, C. Liu et al., Thermally conductive boron nitride nanosheets on electrospun thermoplastic polyurethane for wearable Janus-type fabrics with simultaneous thermal and moisture management. ACS Appl. Nano Mater. **7**, 8229–8237 (2024). https://doi.org/10.1021/acsanm.4c00838
